# Going smoke-free: University staff and students’ qualitative views about smoking on campus during the implementation of a smoke-free policy

**DOI:** 10.1371/journal.pone.0236989

**Published:** 2020-08-20

**Authors:** Marguerite C. Sendall, Chantal Le Lievre, Laura K. McCosker, Lauren Brewis

**Affiliations:** 1 School of Public Health and Social Work, Faculty of Health, Queensland University of Technology, Brisbane, Queensland, Australia; 2 Institute of Health and Biomedical Innovation, Queensland University of Technology, Brisbane, Queensland, Australia; University of Calfornia San Francisco, UNITED STATES

## Abstract

**Background:**

Despite many Australian universities introducing smoke-free policies on campus, there is little information about staff and students understanding of smoking on campus in the context of the implementation of a smoke-free policy.

**Objective:**

This research explores the qualitative views of university staff and students about smoking on campus during the implementation of a smoke-free policy.

**Methods:**

In 2016, an electronic survey was distributed to all current staff and students of a large university in Queensland, Australia during the implementation of a smoke-free policy. The survey consisted of multiple-choice questions about demographics, tobacco use, attitudes towards smoking, awareness of and attitudes towards the policy, and intentions to quit smoking. The final question asked for a short, open-ended response: “Would you like to comment on the issue of smoking on QUT* campuses?*”* This question was extracted from the survey and analysed using inductive thematic analysis. This paper reports the findings from this question. *Queensland University of Technology.

**Results:**

The survey was completed by 641 staff and students. There were 351 responses to the final question. Five inductive themes emerged about smoking on campus during the implementation of a smoke-free policy: 1) the watering down of the policy, if it is not enforced, 2) the creation of hot spots on campus boundaries affecting those who pass by, 3) concern, especially by those who don’t smoke, about the impact on smokers emotional health and welfare, 4) disagreement about the value of designated smoking areas and 5) suggestions about how to better implement the policy.

**Conclusion:**

Overall, participants views about smoking on campus during the implementation of a smoke free policy suggest broad agreement but reflect concerns about enforcement, boundaries, non-smokers and designated areas. Consistent and systematic processes for implementation, maintenance and enforcement of policy goals, and cessation support, is needed to create a non-smoking culture on university campuses.

## Introduction

In 2016, approximately 3 million (14.9%) Australians aged 14 years and over were current daily, less often than weekly or weekly smokers. Of these, 2.4 million (12.2%) smoked tobacco daily [1]. The negative health impacts of smoking tobacco are well-documented [2]. In 2016, tobacco smoking was the leading risk factor contributing to disease in Australia, accounting for 9.0% of the total burden [1]. In 2015, the most recent reporting year, tobacco smoking led to the death of nearly 21, 000 Australians [3]. The related social costs in 2015–2016 are estimated to be $136.9 billion, significantly higher than the $31.5 billion reported in 2004–5 [4]. Similar significant negative impacts associated with smoking are seen internationally [5]. Using a variety of strategies, including smoke-free legislation and policies, bans on advertising, plain packaging, price increases, restriction on sales to minors and public education, Australia has been successful in reducing smoking prevalence [1]. Legislation and policies for smoke-free environments are now commonplace in Australia, and internationally [6]. Since the late-1980s, all Australian states and territories have progressively implemented smoke-free legislation, banning smoking in workplaces and many public spaces such as sporting venues, shopping centres, restaurants, and building entryways [2]. Many organisations, including several Australian universities, have implemented comparable policies. A similar trend towards the implementation of smoke-free legislation and policies has been observed internationally. In 2012, Hyland et al. [7] estimated approximately 11% of the world’s population live in countries with smoke-free legislation and / or policies.

Arguments against smoke-free policies suggest they are not widely supported, are difficult to implement and result in negative impacts such as lost revenue, displacement of smoking from public spaces into the home, and increased stigmatisation of smokers [7]. However, there is a significant body of literature which demonstrates smoke-free legislation and policies result in smoking-related health improvements. International research has demonstrated smoke-free legislation and policies result in significant reductions in smoking rates [8–10]. A meta-analysis published in 2015 suggests smoke-free policies on university campuses specifically result in reductions in smoking rates of 14.7% after 1 year, and 8.3% after 3 years [11]. Smoke-free legislation and policies result in reduced rates of exposure to second-hand tobacco smoke [12]. Research indicates smoke-free policies are generally well-supported, by both staff and students, in university contexts both in Australia and internationally [11, 13].

In 2016, when this study was undertaken, twenty-two Australian universities had introduced smoke-free policies and two had published research reporting the outcomes of these policies [14–16]. This research has been quantitative in nature except for one study [14], which explored reasons for non-compliance. While this study provides valuable feedback for effective policy evaluation, it fails to gain insights from smokers who do comply with new regulations. Staff and students’ views about smoking on campus during the implementation of a smoke-free policy will contribute to a better understanding of compliance and non-compliance and provide evidence-based guidance for policy implementation. This research fills this gap in current knowledge by exploring staff and students’ views about smoking on campus during the implementation of a new smoke-free policy.

## Materials and methods

At the time this study was undertaken in 2016, approximately 5% of universities in the United Kingdom, 25% in the United States [11] and 32% in New Zealand [17] had implemented smoke-free policies. Twenty two out of forty Australian universities had adopted smoke-free policies. Western Australia and Australian Capital Territory had successfully implemented smoke-free policies at all universities. In Victoria, seven out of eight universities have smoke-free policies. New South Wales and South Australia had implemented smoke-free policies at four out of six and two out of four universities respectively. Tasmania and Northern Territory had yet to adopt smoke-free policies at their universities. The remaining eighteen universities still allowed smoking in designated areas or have other unspecified restrictions in place.

At the time, all Queensland universities and other tertiary institutions had smoking policies in place but only two Queensland universities were totally smoke-free. These policies replicate applicable prohibitions outlined in *The Tobacco and Other Smoking Products Act 1998* (the Tobacco Act), such as prohibiting smoking in buildings or enclosed spaces, outdoor eating and drinking areas and near building entrances. Recent amendments to the Tobacco Act could see Queensland universities adopt stringent restrictions regarding smoking prohibitions. The Act suggests public universities in Queensland could act as a government precinct to prescribe regulations where smoking is prohibited in all buildings and grounds.

This research was carried out during the initial stages of the implementation of a smoke-free policy at a large tertiary institution in Queensland, Australia which has nearly 49 000 student enrolments and more than 12 500 staff [18]. The university implemented a smoke-free policy on 01 July 2016. The policy prohibits smoking by all people in all areas of all university campuses, including grounds, buildings and vehicles. This includes the use of cigarettes and all other tobacco-related products (e.g. herbal cigarettes, loose smoking blends, cigarette-making machines, cigarette paper and filters, and electronic cigarettes) [19]. The policy encourages staff, students and visitors to the university to remind others the university is a smoke-free environment including those who they see smoking [20]. The policy specifies ‘disciplinary action’ may be taken in cases of repeated breach of the policy in accordance with relevant Codes of Conduct [19]. The policy directs smokers to areas outside the campus boundaries where smoking is permitted, and to sources of support should they wish to quit smoking [20].

### Participants

An electronic survey was distributed to all current full-time, part-time, casual and sessional staff and all current full-time and part-time, domestic and international students, at all university campuses. The purpose of this survey was to gather information about participants’ (1) current smoking behaviours, and (2) attitudes about the university’s smoke-free policy. The invitation to participate in this survey was emailed during September 2016 via Registrar’s newsletter. The newsletter contained a short introduction about the research and a link to the survey. A follow-up email was sent in October 2016. Survey participants were offered the opportunity to enter a draw to win one of six $50 gift vouchers. Informed consent was obtained from all respondents before data collection. Ethical approval for this project was obtained from the QUT Human Research Ethics Committee (Approval Number: 1600000844).

### Survey

Permission was obtained to use a survey instrument developed and validated by Burns et al. [15] This survey was anonymous and consisted of four sets of focused multiple-choice questions. The first set of three multiple choice questions collected demographic data about the respondents. The second set of questions (six) focused on agreement with smoking attitude statements. The next seven questions asked about agreement with tobacco control attitude statements and the last set of multiple-choice questions (four) focused on the effects of a completely smoke free campus policy. The analyzed data of these questions are reported elsewhere. The final question asked for a short, open-ended response: *“Would you like to comment on the issue of smoking on [the university] campuses*?*”* This question is the focus of this paper.

### Data analysis

The free text responses to the final question were analysed using inductive thematic analysis. A deductive approach based on health behavioural frameworks such as the *Diffusion of Innovation Theory* [21] was considered. An inductive approach was determined to be most appropriate due to the rich and wide-ranging nature of the survey responses.

The process of thematic analysis involved reading each survey response several times to establish familiarity with the data. Most responses were single sentences, many responses were short paragraphs and some responses were long paragraphs. Responses were excluded from the study if (a) the participant did not provide an answer to the question, or (b) the participant’s response was *“no”*, *“nope”*, *“nah”* or *“NA”*. The second and third author, under the stewardship of the first author undertook initial stages of data coding. Significant statements were highlighted, and descriptive codes were generated to reflect the essence of the significant statements in context. This process was repeated three times to ensure codes were sound and trustworthy. The first, second and third authors undertook the later stages of data analysis. Codes were grouped and regrouped to form themes over several months. The themes represent the salient ideas which emerged from the data.

Inter-rater reliability was undertaken by the first and third authors. The process involved cross-checking the allocation of quotes to themes. Twenty-five quotes (approximately 10% of quote pool) were randomly selected to represent data. Identifying codes were removed from quotes. The authors read the quotes and assigned them to the theme which best represented the meaning of the quote. The match percentage was 75% for the first author and 79% for the third author. The authors discussed the mismatched quotes to agree on the theme. Authors did not reach agreement on a small percentage of mismatched quotes.

## Results

The survey was completed by 641 staff and students. Of these participants, 42.9% (n = 275) were staff and 57.1% (n = 166) were students. Most participants were female (74.5%, n = 458). There were 351 responses to the final question. Of these participants, 46.8% (n = 164) were staff and 53.2% (n = 187) were students. Most participants were female (71.5%, n = 251). The percentage difference between survey responses and responses to the final survey question across gender and primary role was 3–4%.

The second section of the survey, *Tobacco use and second-hand smoke exposure* asked respondents about their smoking status. Smoking status can be defined as non-smoker, ex-smoker, regular smoker and occasional smoker [15]. The results of respondents smoking status are presented here to provide context for the findings from the final open-ended survey question. Most participants report they ‘never smoked cigarettes at all, or never smoked them regularly’ (74.1%, n = 260). Approximately one-sixth (15.1%, *n* = 53) of survey participants report they ‘do not smoke now but used to smoke regularly (≥1 cigarette per day)’. Approximately one-fifteenth (6.8%, *n* = 24) report they ‘currently smoke cigarettes (>1 cigarette per day)’, and 3.1% (*n* = 11) report they ‘occasionally smoke cigarettes (on average, <1 cigarette per day)’. Less than 1% (*n* = 3) did not respond to the question about smoking status.

The following five themes emerged from analysing the answers provided to the final, open ended question, *“Would you like to comment on the issue of smoking on [the university] campuses*?*”*: (1) a lack of enforcement, (2) smokers on the boundary, (3) the effects of policy of personal experience, (4) the need for designated smoking areas and (5) suggestions for improving the policy.

### Theme 1 –lack of enforcement

*There is no point having a ban if it’s blatantly ignored*.

Lack of enforcement of the smoke-free policy was identified as a key issue in its implementation. Participants, more so those who do not smoke, felt the lack of enforcement contradicted the implementation of a smoke free policy. Essentially, if the policy was not operationalised to its full extent, it would not achieve its strategic purpose. Participants concluded this because they had not observed university staff enforcing the policy on campus. One participant explained:

*“There are current policies in place*, *however nobody checks or ensures that the policies are being adhered to*. *Hence people just smoke whenever and wherever they want”* (309, female non-smoker).

Participants reflected a lack of enforcement created an environment permissive to smoking. Overall, non-smoking participants, disproportionally to participants who smoke, felt smokers seemed confident to a point of flaunting disregard for the policy. This view was linked to the lack of consequences for disobeying the policy and therefore seen as permission to smoke. Specifically, participants who do not smoke noticed smokers were not held accountable for disregarding the policy and felt enforcement of the rules was fundamental to achieving a smoke free environment. For example, one participant commented:

*“I notice a lot of people ‘getting away’ with smoking on campus*. *For an effective smoke free campus*, *the rules must be enforced”* (230, female non-smoker).

### Theme 2 –smokers on the boundary

A mini gauntlet of thick smoke when entering and leaving the campus.

Participants provided valuable insights into the unintended consequences of implementing a total smoking ban. Effectively, participants thought those who smoke got around the total smoking ban by creating their own ‘designated smoking areas’ just outside the campus boundaries. Consequently, many participants who do not smoke expressed frustration at smokers congregating on the boundary of the campus. These participants explain the clusters of people smoking created large thick plumes of smoke which makes it difficult to avoid passively inhaling smoke. Some participants, predominately those who do not smoke, felt they were more exposed to smoke post implementation of the policy, as one participant explained:

*“…This* [the boundary area where smoking is permitted] *is directly on the main drive when I walk in*. *This means that I have gone from*, *at most*, *once a month exposure due to walking near the smoker's area to*, *at least*, *once a week exposure due to smoker's smoking ‘in’ the botanical gardens but right next to the main drive*. *This is worse as before I knew exactly where to avoid if I did not want to go near smokers*. *Now I don't know when I will be exposed to secondhand smoke”* (242, female non-smoker).

### Theme 3 –effects on personal experience

Butt out of our lives.

Many participants expressed anger about the decision to eliminate designated on-campus smoking areas. Many participants identified a lack of consideration for the impact this could have on the smokers’ personal experience. Interestingly, participants who smoke and those who don’t smoke alike felt a total smoking ban might have unintended outcomes for some people who smoke. Further, some participants who smoke viewed this as impinging on their personal rights to smoke where they choose. Overall, the university was perceived as paternalistic in implementing this type of the smoke-free policy, as one participant argued:

*“[The university] should not be a progressive nanny state*. *Adults should make decisions for themselves*. *Sick of [the university] being a progressive echo chamber for whichever fashionable cause low self-esteem bully's seek to virtue signal with this month”* (194, male non-smoker).

Many participants, mostly those do not smoke, felt strongly about the effect this policy could have on the mental health of people who smoke. In particular, those who do not smoke expressed a genuine and heartfelt concern about the emotional health impacts and the ability to continue studying on those who smoke. This reflected a deeper understanding of the complexities associated with implementing a smoking ban. In fact, participants reflected a total ban could victimise a vulnerable population. Many participants felt sympathetic towards people who smoke. One participant said:

*“I also think the smoking ban unfairly targets people with pre-existing mental health issues as they make up the greater majority of smokers”* (152, female smoker).

Other participants developed this idea further to describe the policy as discriminatory towards people who smoke. Many participants, many who do not smoke, acknowledged difficulties the policy would create for people who smoke, considering there is no smoking area on-campus. Participants felt the University’s hard line failed to recognise smoking as physiologically based and the emotional impacts. Smoking was identified as an addiction which should be addressed through means other than an inflexible policy, as one participant stated:

*“There should be designated smoking areas*. *Smoking is not illegal and it is discriminatory to constantly make it harder for people to exercise their right to smoke by making the entire campus smoke free”* (365, female smoker).

### Theme 4 –designated smoking area’s on-campus

Having a specific area they can go and indulge in their filthy habit away from us breathers.

Most participants, especially non-smokers, were considerate of people who smoke and felt the need for a designated smoking area. A designated smoking area would ensure people who smoke would be confined to areas which others could avoid. Many participants expressed their support for reducing the amount of smoke on campus but there was widespread disagreement between those who smoke and those who don’t about the university’s approach to addressing this. Participants, more likely to be smokers, felt implementing a total ban on smoking was unfair and did not consider the implications for people who smoke. However, participants, more likely to be non-smokers, felt designated areas would be fair and take into consideration the mental health of those who smoke. Participants support the overall goal of the policy but believe designated smoking areas are required for wider support and effectiveness, For example, one participant said:

*“I think it is a great idea to ban smoking*! *However*, *I could imagine that it would be very hard for smokers (particularly staff) as they now have to walk quite a long way until they are allowed to smoke*. *It might be fair to them to still have a designated area that is however away from usual walkways”* (167, female non-smoker).

### Theme 5 –suggestions for policy

What next?

Participants, more likely to be a non-smoker, expressed positive feelings towards the implementation of the policy, and were supportive of the university’s commitment to creating a healthier campus environment. They felt a healthier environment would be good for individuals, the university population and the broader community. Many participants, both those who smoke and those who don’t, emphasised the importance of improving the policy, and provided suggestions. These included: extending the ban, ensuring stronger enforcement, providing counselling to assist people who smoke to ‘quit’, and strategies (including increasing signage) to inform people of the policy. In particular, some participants suggest the smoking ban be extended to include entrance and exit points to the campuses. One participant proposed:

*“Smoking should also be banned at entrances*, *especially from the ferry terminal walkway to the campus and along the stone wall areas towards the Botanical Gardens at Gardens Point*. *It should also cover any areas that would be considered a public access area to all QUT campus”* (287, female non-smoker).

## Discussion

This research provides a unique understanding about staff and students views about smoking during the implementation of a smoke-free policy in an Australian university context. The findings from this research show there is some agreement for the need to introduce a smoke-free policy at the university. This may be a contextual finding due to Queensland’s early adoption of tobacco legalisation. However, the findings of this paper highlight some of the negative aspects of implementing a smoke-free policy such as ineffectiveness. This can be due to enforcement strategies, specifically the lack of enforcement by university staff. This finding is consistent with findings in similar studies involving smoke-free policies on university campuses [17, 22, 23]. Preceding research about smoke-free policies in Australian universities suggests staff do not perceive policy enforcement to be part of their role and are reticent to approach policy violators [16].

Enforcement is critical for the effective implementation of a smoke-free policy. When smoke-free policies are poorly enforced on university campuses, people are more likely to deliberately ignore them [24]. One study from the United States describes a shift towards a more formal approach to enforcing smoke-free policies on university campuses including punitive enforcement strategies such as mandatory participation in prevention classes after policy violation or monetary fines for repeat offenders [25]. However, the extent to which this would be effective and accepted in the Australian context remains unclear. Training opportunities about policy enforcement for staff, leading to a consistent approach to enforcement, have been shown to be effective in other Australian settings such as psychiatric inpatient units [26]. Furthermore, ongoing education and increasing permanent signage may promote increased self-enforcement of the policy [17].

Similar consistent findings to emerge from this research include smokers congregating on campus boundaries, leading to the problem of increased exposure to second-hand smoke for people entering or leaving the campus, and concerns regarding personal safety as many people go to secluded areas to smoke [13, 17]. Procter-Scherdtel and Collins [23] discuss the need to better manage community relations as people who smoke are displaced from university campuses surrounded by residential and shopping precincts. Another significant finding to emerge from this research is the concern regarding the effectiveness of “blanket ban” policies on university campuses. This is consistent with research conducted at other Australian universities which found this policy approach is not widely accepted [13, 14]. However, exposure to second-hand smoke is still a serious aspect to consider when evaluating the overall health of a campus community.

Another downside of implementing a smoke free policy is the impingement of personal freedoms, and the consequences. This research revealed concerns about the effects of a smoke-free policy on personal experience, and smokers’ rights to smoke where they choose. Similar findings are reported in another qualitative study exploring the effects of a smoke-free policy introduced at another large Australian university [14]. This is a finding supported by international literature [27] but challenged by another study which found most people believe the right to breathe clean air should take precedence over the right to smoke and people should only be exposed to harm if they understand the risks and choose to accept them [28].

Lastly, the victimisation of people who smoke by smoke-free policies was another key idea which emerged in this research. Authors Stuber, Galea and Link [29] agree smoke-free policies drive the social unacceptability of tobacco and, subsequently, the stigmatisation of people who smoke. These authors found smoker-related stigma may encourage people to quit or discourage initial uptake. However, the deterrent effects of stigma are not experienced equally across different socioeconomic and racial/ethnic groups, highlighting the need to better understand the stigma processes associated with the smoking epidemic [29]. Ultimately, the role of stigmatisation in the epidemic should be addressed by the tobacco control community to determine whether to promote or discourage this form of deterrence.

### Limitations

There were more non-smokers (n = 251) than smokers who completed the final question on the survey and may reflect a more positive attitude toward the policy. This sample does not have an equal distribution of smokers and non-smokers. Selective non-response and underreporting means non-smokers are underrepresented. A similar study found non-responders were more likely to be people who smoke [30].

## Conclusion

Smoke-free policies have impacted smoking prevalence and exposure to second-hand smoke, but policy implementation may not result in a smoke-free environment. An in-depth exploration of people’s views about smoking on campus during the implementation of smoke-free policies contributes to understanding why, how and under what circumstances smoke-free policies succeed. Our qualitative findings reveal five salient ideas reflecting views about smoking on campus during the implementation of a smoke-free policy; 1) the watering down of the policy, if it is not enforced, 2) the creation of hot spots on campus boundaries affecting those who pass by, 3) concern, especially by those who don’t smoke, about the impact on smokers emotional health and welfare, 4) disagreement about the value of designated smoking areas and 5) suggestions about how to better implement the policy. However, successful ecological approaches to tobacco control rely on consistent and systematic processes for implementation, maintenance and enforcement. Clear and frequent communication, education, regular and ongoing surveillance, permanent signage, positive reinforcement and tobacco cessation support services are essential during this transition period to assist in establishing a culture of compliance for a smoke-free environment.
